# Representing Animals: Moral and Epistemic Limits for Protection Against Cruelty

**DOI:** 10.3390/ani14213112

**Published:** 2024-10-29

**Authors:** Luís Cordeiro-Rodrigues, Demin Duan

**Affiliations:** 1 Department of Philosophy, Yuelu Academy, Hunan University, Changsha 410082, China; 2 School of Government, Peking University, No. 5 Yiheyuan Road, Beijing 100871, China

**Keywords:** animal cruelty, animal voting, basic rights, representatives, tyranny of the majority

## Abstract

Some philosophers argue that animals should be included in the democratic system, with people acting as their representatives in voting on issues that concern them. This article contends that, while animals’ rights are fundamentally important, granting people rights to represent animals in democratic processes may lead to the opposite of what we want. Or worse, it may put animals’ interests and rights at significant risk. If animals’ rights are basic and straightforward, as proponents of this proposal assume, then deliberation is either redundant or dangerous in safeguarding the interests of animals.

## Simple Summary

This paper rejects the argument that granting people the right to represent animals in democratic processes is a morally desirable option. We uphold that this may lead to more violations of animals’ rights and recommend a different approach.

## 1. Introduction

Some philosophers forward the intriguing proposal that animals should be included in the democratic representative system and have some voting rights regarding limited topics [1,2,3,4,5,6]. Notably, the system sometimes proposed is to have representatives voting on behalf of animals concerning a small group of topics that affect the well-being and interests of animals [1,2]. In this article, we reply to the proponents of this proposal. We do not aim to answer all possible arguments in favour of animal voting through a representative system. This aim would require an extensive overview of a democratic theory, which is unfeasible for the size of an article. Instead, we want to reply to a specific kind of argument in favour of voting rights for animals in a representative system: the argument that animals should have voting rights because they can also reveal preferences and interests, and, given that real democracy requires the inclusion of all, then animals should be included in the voting process with the help of representatives. In reply to this perspective, we contend that this proposal fails to guarantee that animals’ interests are represented and puts animals’ interests and rights at significant risk. If animals’ rights are basic and straightforward, as proponents of this proposal assume, then deliberation is either redundant or dangerous in safeguarding the interests of animals.

The problem is not simply that including animals in the democratic process has challenges. Indeed, some of these challenges are like other challenges faced by any democratic process, and they are insufficient to reject democratic processes. One of the shared challenges is, for example, the fact that media and social prejudice can influence democratic outcomes. Even with this challenge, the issue of including animals in voting is more complex for two major reasons, which show that the proposal of giving voting rights to animals should be rejected. Firstly, representatives cannot meaningfully and truthfully represent the interests of animals. Secondly, because animals’ interests are basic and straightforward, to include a deliberative process to forward their interests is either redundant (it does not add anything to what we already know) or can potentially allow political opportunism and put at risk the interests of animals. Note that we do not necessarily agree that animals’ interests are simple. Instead, this statement is the premise that those defending this proposal start from, and they need this premise to make their argument. For if animal interests are considered complex, then the arguments they put forward in favour of animal voting fail (as we argue later in this paper). To clarify, as we would not vote for basic rights for humans (such as the right to life), we should also not vote for the basic rights of animals. What is being voted on in the case of animals are rights like this because animals’ interests are basic; thus, the deliberative process does more harm than good, defeating its initial purpose. Furthermore, contrary to what the proponents of this voting proposal contend, the problems found in animal voting are not the same as the ones found in human voting. So, there are good reasons to reject animal voting rights.

We divided this article into four sections. The first section describes the kind of cruelty that animals suffer and the challenges we face to address this cruelty. The purpose of this section is to explain the context and motivation that the proponents of the proposal for having representatives for animals have for extending political rights to animals. Precisely, the context is that animals have interests that matter, these interests are routinely violated, and animals still suffer significant cruelty today. Hence, more strategies are needed to address this. As a result, they recommend the use of a representative system as a solution. Although we do not think the representative system is a good solution, we recognise that more needs to be done to protect animals from cruelty today. Section Two outlines the case for giving animals the right to vote arguments. In Section Three, we demonstrate how this proposal fails to be convincing and brings more problems to protecting animals’ interests. Section Four addresses some potential objections we have overlooked regarding the proposal’s value for having representatives for animals. We reply, pointing out some moral limits of representation and raising the issue of tyranny of the majority. More precisely, they may respond that the issues we pointed out also exist in other voting systems; however, we dispute that the case of animals is not the same as the problems we find in electoral systems.

## 2. The Context for the Proposal: Persistent and Pervasive Animal Cruelty

The motivation of the proposal for animal voting is to address the neglect of animals’ interests. This neglect of animals’ interests is understood here as animal cruelty. Thus, it is critical to understand this concept. Animal cruelty has several dimensions [7,8]. It has a behavioural dimension that involves acts that cause unnecessary physical or psychological distress, pain, or suffering, or death of the animal. Another dimension is the target victim. Specifically, such suffering must be inflicted on a sentient being [9]. The act can be both illegal or legal, given that its critical feature is not whether it is socially acceptable or institutionally legitimate but whether it inflicts the kind of suffering aforementioned. The concept of ‘animal cruelty’ refers to the type of behaviour that disrespects the fundamental interests of animals, so it does not matter if it is socially or legally acceptable, but whether it causes unnecessary harm. It involves treating animals below a threshold of minimum acceptability of how those animals must be treated, given that they are capable of suffering. It is, therefore, a form of inflicting suffering on animals. There are two primary categories of animal cruelty: abuse and neglect. Abuse involves intentional harm to the animal, and it can include actions such as mutilation and denial of access to food and water. Neglect refers to the failure to provide for the needs of animals. This action does not need to be intentional, but it involves treating animals in a way that is inadequate for them and causes them suffering. For example, it is neglectful to give chocolate to a dog, even if we do not know that this may cause the dog’s death [10].

The proposal’s proponents note that respect and protection of animal interests are very far from being met today [1,2]. Unfortunately, in the contemporary world, animal cruelty is a widespread reality that is present in many areas of life. To recall, we do not understand animal cruelty in this article simply as illegal and socially unacceptable practises; instead, our understanding of animal cruelty is animal-centred, focused on the unnecessary suffering of animals. Four paradigmatic examples of animal cruelty in today’s world are factory farming, animal testing, wildlife tracking and poaching, and animal entertainment. A practise where animal cruelty is significantly widespread is in industrial factory farming. Routinely, concerning massive-scale farming, animals are routinely confined to unhygienic conditions in overcrowded places to maximise the production of meat and dairy products. Such animals are kept in small cages with little room to move, and their movement and behaviour are highly restricted. This practise has a strong negative impact on their physical and psychological health [11,12,13,14]. Furthermore, often, slaughtering practises are inhumane and cause unnecessary and prolonged pain [15,16,17,18,19]. Mass industrial farming also increases the transmission of viruses between different species for several reasons. To start, transmission of viruses is facilitated in overcrowded spaces. The aforementioned induced stress also damages immune systems, making animals more vulnerable to viruses. On top of this vulnerability, it is the inadequate sunlight and ventilation conditions of many industrial factory farms that helps spread such viruses [11,12,14]. Note that although other kinds of production of meat and dairy may be ethically objectionable, the description we make above refers only to industrial farming and not other kinds of farming. Simple, natural, free-range systems are different from industrial farming.

Another way animals suffer significantly is through product testing on animals, especially the testing of cosmetics and drugs. Many cosmetic companies today try their products first on animals, especially rabbits and mice. This practise causes a great deal of skin and eye irritation to animals, and, in some cases, the testing may be lethal to the animals [20,21]. But it is important to note that 45 countries worldwide have banned totally or partially testing cosmetics on animals [22]. Additionally, animals are routinely used for medical and drug testing experiments. A conservative estimate of the number of animals used worldwide for research is 58.3 million, but if we include different kinds of laboratory use, the number may rise to 115.3 million animals [23]. The animals normally used for this laboratory practise include mice, rats, different kinds of rodents, pigs, dogs, cats, fish, amongst others [23,24,25,26]. Most, if not all, laboratory procedures cause great distress to animals [27,28]. For example, mice and monkeys used in labs routinely show signs of distress [29,30]. It is also common for animals to suffer permanent injuries, such as defects in their hearts and damage to blood vessels [23,31,32]. These injuries involve, for example, toxic testing and invasive surgeries, which cause a great deal of suffering, pain, injury, and death [20,21,33,34,35,36].

Another common form of animal cruelty is wildlife trafficking and poaching. Wildlife crime is incredibly profitable, estimated to be worth US $8–10 billion every year [37]. Routinely, many wild animals are illegally captured, killed, and sold. Specifically, this crime may occur to sell some of their body parts, like their horns, fur, and bones, but it can also be a form of exotic pet trade. Elephants, rhinos, and tigers are common victims of poaching, as their body parts are often considered monetarily and symbolically valuable. In the case of rhinos, due to poaching, the number of this species left in the wild is only around 25,000 [37]. Monkeys, parrots, turtles, and reptiles are sometimes captured to be used as exotic pets [37]. They endure a significant amount of stress, are taken out of their natural environment, are carried out in inhumane and inadequate transportation conditions, and are given inadequate care [38,39,40]. In addition to the direct harm made to animals, poaching and kidnapping makes ecosystems more fragile, which, in turn, reduces biodiversity and hurts other animals and the environment [37].

Animal cruelty in animal entertainment can be found in circuses and animal shows, zoos, and animal fighting. In circuses, many animals, like elephants and lions, suffer harsh punishments, are kept in small spaces and are transported under inhumane conditions. In zoos, routinely, animals are taken from their natural habitat and placed in unnatural enclosures with little stimulation. In some places, some people organise bullfights, dogfights and cockfights. Such sports involve different kinds of harm to the animals, such as pain, death, harsh training, starvation, and injuries [41,42,43,44].

Why is this relevant to the proposal for animals’ representatives? The very fact that cruelty towards animals is widespread indicates that there is a lot to do for animals. Particularly, let us look at the history of animal cruelty legislation. It is clear that although this legislation has existed for a long time, it has not done enough to protect animals’ interests. Cruelty was generally accepted as part of God‘s plan in the Middle Ages [45]. In the Renaissance, there were concerns about changing this idea, and concepts of anti-cruelty started emerging, but they were primarily focused on humans. With the Enlightenment, concepts of cruelty applied to animals emerged. Philosophers like Immanuel Kant and John Locke showed concerns about it [46]. Also, during this period, the British artist William Hogarth produced an artwork entitled ‘The Four Stages of Cruelty’, depicting how someone cruel to animals may eventually become cruel to humans.

A bit later, in the 19th century, the philosopher Jeremy Bentham, in his *An Introduction to the Principles of Morals and Legislation*, suggested legislation to protect animals [47]. Indeed, the first animal protection laws emerged in the 19th and 20th centuries. Lord Eskine wrote in his ‘Cruelty to Animals Bill‘ the following:

“*The abuse of that [human] dominion by cruel and oppressive treatment of such animals, is not only highly unjust and immoral, but most pernicious in its example, having an evident tendency to harden the heart against the natural feelings of humanity*”.

(cited in Linzey, 2009; p. 1)

This bill proposed by Lord Eskine was passed in the House of Lords but failed to pass the House of Commons. But, in 1822, Richard Martin was able to pass a similar bill known as “Dick Martin’s Act” [48]. The legislation protecting animals emerging primarily in the UK and then in the US was focused on the violation of property and not so much on animals themselves. An example is the Vermont legislation, which protected animals by making damaging other people‘s property illegal. However, at this time, there was no provision prohibiting animal cruelty. However, such animal anti-cruelty laws emerged in the late 19th century and early 20th century. The concepts did not develop alone. At the same time, legislation for abuse towards women and children has also grown [48]. Today, cruelty is also a term used in International Humanitarian law.

The law has developed significantly worldwide since. Most countries have animal anti-cruelty legislation [49,50,51,52]. However, it may not encompass all animals and may be used flexibly. For example, poultry is sometimes excluded from animal legislation or has separate legislation that allows significant amounts of cruelty. Likewise, sometimes, the law’s definition of ‘animals’ deliberately excludes farm animals to allow a different treatment [49]. Likewise, many laws still focus on the property paradigm, which does not sufficiently protect the animal [53]. Some animal movements were accused of misrepresenting the real interests of animals [18,19,54]. *The point is that despite the relatively long history of legislation*, as this section demonstrates, animals still suffer significant harm today. Animals have interests that are neglected by legal and illegal industries. Today, there exists a pervasive and persisting animal cruelty. Therefore, a new way to deal with animals is necessary and urgent. The proposal proponents discussed in this article note this phenomenon and are concerned about it; they want to advance a strategy to address this failure. In the next section, we will explain more details about the proposal. Still, later, we will show that, even though we must be worried about animals, having representatives is not a good way to protect animals.

## 3. Representing Animals

The idea of representing animals is becoming increasingly important. The Dutch parliament has a Party for Animals campaigning to advance animals’ interests [55]. Likewise, in the Portuguese parliament, the party People, Animals, and Nature (formerly Party for Animals and Nature) has proposed several policies to advance animals’ interests [56]. As Robert Goodin has pointed out, although the concerns for animals and the environment are legitimate, the political theory underlying it is not sufficiently developed and sophisticated [4,57]. In other words, there are legitimate concerns about animals, given how animals are treated and defending such interests is morally urgent. However, these movements’ discourses lack a convincing moral justification of why this is the case. Hence, some philosophers have tried to advance ethical and political theories that can justify representing non-human interests, including animals [1,2,3,4].

The case for animal voting is not to imagine animals voting themselves. Indeed, such an approach would be ridiculous, given that most animals (potentially all) would not understand the action. The idea is grounded instead on the premise that human proxies can represent animals in some situations, similar to how adults represent children. Goodin, for example, links the proposal with the idea that parents are usually understood as trustees of their children’s interests [4].

Instead of the incoherent proposal involving direct voting by animals, these scholars suggest a representative system to institutionalise animal voting rights. Their proposals have the same core ideas but are slightly different in some respects. Goodin advocates for representatives becoming the trustees of nature when engaging in deliberation [4]; Dobson, similarly, considers that these representatives could be trustees and delegates for animals and future generations [2]. Christopher Stone contends that due to the moral standing of nature, it must be given legal representation [5]. Tine Stein proposes an Ecological Council to protect nature’s interests [58]. Despite their differences, in all proposals, the key point is to forward a system where representatives would speak on behalf of non-humans (either nature or other animal species). These representatives must be submitted for re-election [1,2,4].

The representatives of animals would be constituted by a politically neutral committee with scientific experts appointed by multi-partisan agreement. They would have a similar role to that of guardians. Representatives would not have the right to vote on topics such as bankruptcy or corruption policies, which do not concern animals. When it comes to more controversial issues, like environmental policies, which may impact the welfare of animals, it is optional to include animals, at least until we can have a robust scientific consensus about these issues or leave animals out [1]. Other themes that are more straightforward and consensual must be voted on.

But what exactly morally justifies such a proposal? To answer this question, we must return to the previous section. As it is clear, animals’ interests are being violated, and a way to protect them is needed. Underlying this idea, however, there is a more fundamental one: namely, that animals do have interests. As Dobson contends, ‘Long ago animal rights theorists breached the species divide in one direction by pointing out that, whatever characteristic was chosen to distinguish humans from animals, that characteristic would be found to be possessed by some animals as well as all humans.’ [2]. More fundamentally, if animals have interests they also have rights: ‘Either way, the human/other species divide is porous: if humans have rights (including political rights) then at least some animals have them too.’ [2].

But if animals have rights like humans, how does this premise extend to understanding that they have political rights? The grounds for understanding that they have political rights is because, as Robert Dahl has stated, ‘Everyone who is affected by the decisions of a government should have the right to participate in that government.’ [59]. If we agree to some version of this principle, we must also include animals in the participation of government, for as we noted, animals do have interests; therefore, they must also participate in government. Alfonso Donoso most eloquently explains this as follows:

  i. X has moral standing if and only if x has relevant interests;
 ii. If x has relevant interests, then x deserves legal and political representation;
iii. Non-humans have relevant interests;

Therefore:

 iv. Non-humans have moral standing.
  v. Non-humans deserve legal and political representation [3].

In short, if some version of this principle is true, it implies that some animals should have the right to participate in government, for it is undeniable that animals have some level of interests.

The initial reaction may be that animals are not competent to vote, so the proposal must be dismissed. But this exaggeration misses the fact that animals also have agency. Animals can show agency in many ways [6,60,61]. We must pay attention to how animals are signalling their preferences and try to understand that the expression of preferences does not need to be human-centric [60,62]. If we decentralise from a human-centric perspective to express preferences and interests, we can see animal behaviour as expressing what they wish. Animals can express themselves through less sophisticated themes. If this is the case, as long as animals can express their preferences on some topics, they must be included in voting processes [1,2,3,4]. The core of this argument is grounded on the premise that there is something evident about what animals’ preferences/interests are. The primary reason is that animals’ interests are straightforward and obvious, and we can detect their preferences by interacting with them. Take, for example, Motoarcă’s take on this topic:

‘*The fact that animals’ interests can be tracked very well by the voting proposal under discussion is good reason to believe that the latter is a legitimate voting procedure, independently of any competence considerations.*’

[1]

‘*What ultimately makes this entire voting arrangement possible and sensible simultaneously is that animals’ interests are fairly straightforward compared to people’s. Animals do not worry about complex moral problems like abortion or the legalisation of prostitution, so these are not appropriate topics for animals to vote on.*’

[1]

From this perspective, there is no gap between preferences and interests in the case of animals. According to these authors, animals do not have existentialist and complex feelings or moral concerns; therefore, what interests them is straightforward. There may be some kind of slightly more complex feelings, but they are fundamentally basic and primary.

## 4. Epistemic Limitations of Representatives for Animals

One critical reason why the pro-voting rights argument fails to provide sufficient reasons for animal voting rights is that the multi-partisan elected representatives cannot meaningfully and trustworthily represent animals. This trumps the purpose of giving voting rights to animals. Other things equal, it is morally acceptable for representatives to substitute representees when the following conditions are met:

(1) The representee, for some reason, cannot represent herself (This point is more complex than stated but for the current purpose it suffices).
(2) There is a strong connection between the representative and the representee so that
  (2.1) We are sure/confident that the representative acts in the best interests of the represented
  (2.2) The representative knows the representee sufficiently well to be able to give voice to the latter’s interests and opinions.

The two following examples illustrate this concept well. We allow parents to make decisions for their children because we assume that children cannot always express their interests well (1), the parent–child relationship is usually sufficiently strong (2) to think that they act for their children’s best interests (2.1), and they know their children well enough to choose for them (2.2). In cases of assisted death where the patient cannot express their interests (1), often the closest people (parents, partner, etc.) are the ones who are most likely to have the best interest of the patient (2.1) and to know the patient better (2.2) due to the connection they have (2).

Now, the case of representatives of animals is radically different from these two examples. There is no convincing way to determine whether representatives have a strong connection with animals, know them well enough, and are meaningfully and trustworthily acting in their best interests. It is difficult to identify who would be the actual representative of animals. Representatives are not family or friends of animals; they become representatives by a multi-partisan vote. However, different sectors of society may claim to be specialists in animals’ interests and, thereby, be the best representatives of animals. For example, in Portugal, bullfight practitioners claim to be the real representatives of animal interests. They argue that the bull does not feel pain and, instead, feels excitement with the bullfight. They claim it is the nature of the bull to fight, and they are the ones who respect the bull for this reason [63]. Although this argument may sound absurd to some, the argument is convincing for many. Similarly, factory farmers and researchers who carry out animal testing could claim that they are really representative of animals’ interests, according to the voting proposal.

More problematically, there is no objective way to identify who really must be the representative: it is either needed to select a list of interests to which the representative must pay attention, or it is necessary to vote democratically. If the proponents of animal voting rights accept that a list of interests to be respected must be provided in advance, they are undermining their system for two reasons. For one, they enter into self-contradiction because they reject the idea that these ideas should be decided outside a democratic space, so there is no reason to open an exception here. Additionally, the representative’s role becomes redundant as the interests have already been agreed beforehand. This redundancy is especially the case because the proponents of the voting proposal consider animals’ interests to be straightforward and basic, and therefore, deliberation seems unnecessary.

If, instead, everything must be voted (or not necessarily everything, but just complex interests must be voted, with a pre-agreed set of fundamental interests), it is unclear how this action can meaningfully and trustworthily represent animals. Unlike the case of parents or surrogates, there are too many political interests at stake, which, therefore, allows political opportunism and undermines the neutrality that the proponents of the voting proposal ask for the process to go well. In a non-ideal world, where corporations have interests in influencing the public and ag-gag laws curtail speech about such corporations [64], elected representatives seem unlikely to be politically neutral to represent animals authentically.

Furthermore, a majority vote on representatives is more likely to reflect the prejudice of the majority towards minority practises that involve animals and some animals. Routinely, minorities have practises that are disapproved of by the majority (such as ritual slaughtering, eating dogs, etc.) [65,66]. Objectively, however, there is little difference in the harm made to the animal by many majority and minority practises. Take the example of dog eating: there is no good moral reason not to eat dogs if we think it is morally acceptable to eat pigs or cows. Voted representatives will represent the majority more than the minority, mirroring such prejudices, reinforcing majority practises, and disallowing others. The process of voting allows institutionalising prejudice and discrimination towards minorities.

Regarding prejudice towards some kinds of animals, note that, as Sue Donaldson and Will Kymlicka have pointed out, liminal animals are often victims of prejudice and discrimination. Liminal animals are those that opportunistically live amongst human communities, can unlikely adapt to the wild, and cannot be domesticated, such as rats, sparrows, etc. Liminal animals are usually not welcomed by humans and are often considered vermin [60]. Individuals typically disregard these animals and consider them inferior to other animals. Given widespread prejudice against such animals, the elected representatives are likely to mirror these societal prejudices, and such animals would most likely suffer from further prejudice. Put differently, if humans were to vote for the rights of liminal animals, these animals are unlikely to be protected, and the voted policies are more likely to reflect human prejudice. Such animals seem much better protected with a set of rights that guarantees their protection.

## 5. The Moral Limits of Representation and Tyranny of the Majority

The proponents of the voting proposal may object to our argument that unfit representatives, undue political influence, societal prejudice, and adverse outcomes, in general, are problems that exist in democracy, and none of them is a sufficient argument against the democratic process. Our arguments demonstrate that including animals in voting causes more harm than good. However, the proponents of the voting proposal could argue that this kind of argument can only be accepted if it is based on empirical evidence, and I have not presented any [1]. Additionally, there is no legitimate judge of the quality of the outcome to determine this question: who is to decide?

We should start by replying that the argument does not require empirical evidence to be rejected. That is an impossible criterion for refutation because, to date, no electoral system has included animals. Fortunately, it is also unnecessary because we need a reasonable justification for not giving voting rights to animals. Philosophical argument is grounded on justification, and this justification does not need to be empirical. The critical question, however, is why democracy tends to be chosen over other systems. The reason is that democracy, other things equal, tends to be the best way to identify and provide what people want, even if the consequences of the democratic process are harmful, even if there is political influence, and so forth. The point is that the value of democratic processes is instrumental in expressing preferences. If it fails to accomplish this action, it fails one of its fundamental purposes. Thus, democratic processes are not used simply because they are unconditionally or intrinsically valuable: if we were to vote for killing everyone from a specific ethnic group, such a voting outcome would not be more valuable than an authoritarian regime prohibiting such killing. Democratic processes are valued for what they can offer. Nonetheless, it is unlikely that voting is the best option for understanding and respecting the interests/preferences of individuals, like animals, who cannot represent themselves. So, the question is whether representatives can really express or at least improve the identification of preferences of animals and forward, thereby, animals’ interests.

A clarifying way to think about how the proposal is problematic is to imagine that we had a committee of white males representing women. They would go to parliament, and after surveying women’s preferences, they would make laws about them. The example feels uncomfortably familiar as this is the form of legislation that existed before women’s voting, and no reasonable person today thinks it is legitimate. The reasons why it is not legitimate are varied, but there are at least two fundamental ones. Firstly, there is too much conflict of interest, which obviously will curtail the legitimacy of the voting. Secondly, the person herself is the best evaluator of their condition, and no one can fully replace them. The problem is that the purpose of including animals in voting is defeated because representatives cannot represent animals’ preferences well. Having a committee with so much power is as worrying as having a group of white males representing women.

The proponents of the voting proposal can push back that it is a different situation because women can represent themselves, but animals cannot. However, the argument would also not work for children or people in a coma, who are more like animals regarding the capacity to self-represent. The right people to represent children are their parents, and the right people to represent people in a coma are their close significant others because of the proximity they have; if we were to have a group of elected representatives to vote for what our children should do or to claim to represent all the people in a coma, we would feel the relationship is too distant to make sense and for decisions to be meaningful. The critical difference is the proximity to the representee, how this proximity is relevant to meaningful representation and how easy it is to get it wrong.

The proponents of the voting proposal can also reply that this meaningful representation is still a problem for all democracies, and, in this case, the best thing to do is still to get the representatives; that is better than nothing. Goodin, who endorses having representatives for animals, recognises that the option is the second best, given that animals and nature cannot represent themselves. Still, it is better than nothing [4]. However, this does not seem to be the case. Given that the primary purpose of giving voting rights is to benefit animals, it is difficult to see how it is better than nothing because no unique benefits would come from voting. Hence, the interests of animals would be better served by constitutional rights than voting. To make an analogy, the rights of the LGBTQ+, such as protection from violence, the right to marry, and the abolition of laws that condemn same-sex relations as a crime, are not necessarily better served by voting representatives who would speak on behalf of the LGBTQ+; instead, it is by having robust constitutional protection against violations of those rights that the rights of the LGBTQ+ would be protected. Imagine, for example, in a highly homophobic society that we were to vote on whether same-sex relations were a crime, and it could be made on grounds of being of the interest of homosexuals to ‘cure themselves’ from ‘disease’. This has no benefit, and introducing voting can worsen their situation. Referendums are sometimes strategically used as a tool precisely to curtail the possibility of advancing progressive policies. Appertaining a similar line of reasoning, we do not live in a post-speciesist society, and therefore, if animals’ interests are voted for, the likely outcome is to reinforce speciesism.

This kind of problem that democratic processes can bring up outcomes that are undesirable and unfair to certain groups can be traced back to Plato’s *Gorgias*, where Callicles states the following: “The makers of laws are the majority who are weak; and they make laws and distribute praises and censures with a view to themselves and to their own interests” [67]. This problem of democratic processes is often called ‘the tyranny of the majority’. The term’s origin can be traced to Alexis Tocqueville in his *Democracy in America* [68]. Tocqueville observes that in a society of equal individuals, It is very difficult for anyone to resist the majority’s will. But the majority’s opinion is not always right; actually, very often, it is the opposite. John Adams and James Madison also widely used the term to advocate for government limits [69,70]. John Stuart Mill, however, is the one who developed it further [71,72]. Mill’s fundamental concern was with legislatively enacted restrictions of liberty, but he also worried about informal mechanisms of social pressure. Mill recommended that, roughly speaking, liberty can only be restricted to prevent harm to others. Mill’s concern is not liberty per se but to defend *fundamental liberties*.

Although we do not want to defend the harm principle here, we want to point out that, as Mill and others noted, relying too much on democratic processes may lead to oppressive outcomes that violate individuals’ fundamental liberties. So, constitutional guarantees may preserve individuals’ fundamental liberties better than deliberative ones, even for someone like Mill, who believed that representative democracy was the best political system [72]. It is possible to recognise that representative democracy has many benefits, but at the same time, it must be balanced with a concern for individuals’ rights and liberties. Fundamentally, as Robert Nozick and Ayn Rand contend, individual rights must not be subject to public vote, and the point of rights is precisely to protect individuals from oppression. We do not need to be libertarians like Rand and Nozick to recognise this fact: it is sufficient to acknowledge some aspects of one’s life that others must not rule. Precisely, in the case of animals, given that their interests correspond to basic/fundamental rights (as the proponents of animals’ representatives themselves recognise), deliberation either adds nothing to it or puts these fundamental rights into discussion, risking legislating against such rights [73,74].

For most animals, except those who cannot survive by themselves, the best course of action, other things being equal, is to leave them alone rather than voting for, say, whether their cages should be 10 or 15 square metres. For the most part, animal suffering primarily comes from human interference, and therefore, to place humans to further deliberate on animals’ interests is a bad idea. Additionally, most interests at stake are straightforward and basic. By this, we mean that animals’ interests are evident and very rudimentary, such as not being harmed, not being killed, and being able to drink water and eat. Resultantly, these interests can be included as part of a constitutional non-harm principle and a right-to-life principle that protects these rudimentary interests without the need for deliberation.

## 6. Conclusions

This paper has contested that animals should have representatives of their interests who would vote on their behalf. Our key argument is that representatives cannot meaningfully and trustworthily represent animals. Furthermore, democratic processes are limited in protecting individuals’ interests and may lead to the tyranny of the majority. This, in turn, implies that the interests of animals are at risk of being curtailed by a voting process. Additionally, we argued that animals’ interests are straightforward, rendering the democratic process redundant. All in all, animals’ interests would be better served with a constitutional system guarantee.

## Data Availability

No new data were created or analyzed in this study. Data are contained within the article.
